# Knock-Down of Endogenous Bornavirus-Like Nucleoprotein 1 Inhibits Cell Growth and Induces Apoptosis in Human Oligodendroglia Cells

**DOI:** 10.3390/ijms17040435

**Published:** 2016-03-24

**Authors:** Peng He, Lin Sun, Dan Zhu, Hong Zhang, Liang Zhang, Yujie Guo, Siwen Liu, Jingjing Zhou, Xiaoyan Xu, Peng Xie

**Affiliations:** 1 Department of Neurology, Yongchuan Hospital, Chongqing Medical University, Chongqing 402460, China; hepeng000@sina.com; 2 Chongqing Key Laboratory of Neurobiology, Chongqing Medical University, Chongqing 400016, China; tilamisu789456123@126.com (L.S.); zhudan25@126.com (D.Z.); ASDFG43215@126.com (H.Z.); zhlbright@gmail.com (L.Z.); g1240725344@163.com (Y.G.); 18983145528@163.com (S.L.); duduzjj@163.com (J.Z.); xxy2922@163.com (X.X.); 3 Institute of Neuroscience and the Collaborative Innovation Center for Brain Science, Chongqing Medical University, Chongqing 400016, China; 4 Department of Neurology, the First Affiliated Hospital, Chongqing Medical University, Chongqing 400016, China

**Keywords:** endogenous bornavirus-like nucleoprotein 1, RNA interference, cDNA array, cell proliferation, apoptosis

## Abstract

Endogenous bornavirus-like nucleoprotein elements (*EBLNs*) have been discovered in the genomes of various animals including humans, whose functions have been seldom studied. To explore the biological functions of human *EBLNs*, we constructed a lentiviral vector expressing a short-hairpin RNA against human *EBLN1*, which successfully inhibited *EBLN1* expression by above 80% in infected human oligodendroglia cells (OL cells). We found that *EBLN1* silencing suppressed cell proliferation, induced G2/M phase arrest, and promoted apoptosis in OL cells. Gene expression profiling demonstrated that 1067 genes were up-regulated, and 2004 were down-regulated after *EBLN1* silencing. The top 10 most upregulated genes were *PI3*, *RND3*, *BLZF1*, *SOD2*, *EPGN*, *SBSN*, *INSIG1*, *OSMR*, *CREB3L2*, and *MSMO1*, and the top 10 most-downregulated genes were *KRTAP2-4*, *FLRT2*, *DIDO1*, *FAT4*, *ESCO2*, *ZNF804A*, *SUV420H1*, *ZC3H4*, *YAE1D1*, and *NCOA5*. Pathway analysis revealed that these differentially expressed genes were mainly involved in pathways related to the cell cycle, the mitogen-activated protein kinase pathway, p53 signaling, and apoptosis. The gene expression profiles were validated by using quantitative reverse transcription polymerase chain reaction (RT-PCR) for detecting these 20 most-changed genes. Three genes closely related to glioma, *RND3*, *OSMR*, and *CREB3L2*, were significantly upregulated and might be the key factors in *EBLN1* regulating the proliferation and apoptosis of OL cells. This study provides evidence that *EBLN1* plays a key role in regulating cell life and death, thereby opening several avenues of investigation regarding *EBLN1* in the future.

## 1. Introduction

Up to 8% of the human genome is comprised of genetic material from human endogenous retroviruses (HERVs), which originated from the integration of retroviral DNA into chromosomes of germline cells and subsequent inheritance in offspring [1]. Although most HERVs are inactivated or silenced by mutations or epigenetic modifications, they have served important functions in human evolution and speciation, and can potentially cause or contribute to diseases [2,3]. Mounting evidence has demonstrated that HERVs may be involved in the pathological processes of some neurological and psychiatric disorders, such as multiple sclerosis, schizophrenia, and bipolar disorder [4,5], and cancers such as melanoma, breast, prostate, and leukemia [6,7]. Thus, investigating HERVs is important for understanding the etiological mechanisms of certain diseases.

Retroviruses are thought to be the only viruses that generate genomic HERV DNA insertions. Recently, sequences highly homologous to the nucleoprotein (N) gene of bornavirus, a non-retrovirus, were found in the genomes of several mammalian species, including the human genome, and designated as endogenous bornavirus-like N (EBLN) elements [8]. Bornavirus is a non-segmented, negative-sense RNA virus that is characterized by persistent infection in the cell nucleus [9,10]. Borna disease virus (BDV) is a mammalian bornavirus of the *Bornavirus* genus in the *Bornaviridae* family. BDV can infect many vertebrate species, including humans [11,12,13,14,15,16,17]. The BDV genome is approximately 8.9 kb long and contains 6 open reading frames (ORFs) encoding N, phosphoprotein (P), X protein (X), matrix protein (M), glycoprotein (G), and polymerase (L) [18]. BDV N is a major structural protein that serves an important role in the formation and transport of ribonucleoproteins [19,20,21].

Previous results showed that rodent *EBLNs* might play an important role in BDV infection. Species containing *EBLNs* could be protected against circulating bornavirus [22]. Similarly, *EBLNs* in the genome of the thirteen-lined ground squirrel could efficiently inhibit infection and replication of extant bornavirus by regulating the activity of the BDV polymerase [23]. Recently, Parrish *et al.* [24] reported that *EBLNs* can give rise to PIWI (P-element induced wimpy testis)-interacting RNAs (piRNAs), a class of small RNAs known to silence transposons, engendering a RNA-mediated, sequence-specific antiviral immune memory. Nevertheless, the functions of *Homo sapiens* *EBLNs* are still not well known.

To date, a total of seven *EBLNs* have been found in the human genome [25]. The *EBLN1* gene shows up to 58% similarity to the nucleotide sequences of BDV *N* gene, and contains a long ORF encoding a potential protein of 366 amino acids. Although the evidence of EBLN1 protein expression is lacking, *EBLN1* mRNA expression has been confirmed by reverse transcription polymerase chain reaction (RT-PCR) in several cell lines including OL, HEK293T, and MOLT-4 cells [8,25], suggesting that *EBLN1* might be a pseudogene or function as a noncoding RNA.

Here, we report that *EBLN1* silencing by short-hairpin RNA (shRNA)-expressing lentivirus could inhibit human oligodendroglia (OL) cell proliferation and induce apoptosis. Furthermore, the gene expression profiles of OL cells after *EBLN1* knockdown were analyzed using a cDNA microarray. Our work will expand the field of functions of *EBLN1* gene.

## 2. Results

### 2.1. Effective Reduction of Endogenous Bornavirus-Like Nucleoprotein 1 (EBLN1) mRNA Expression with an shRNA

**Figure 1:**
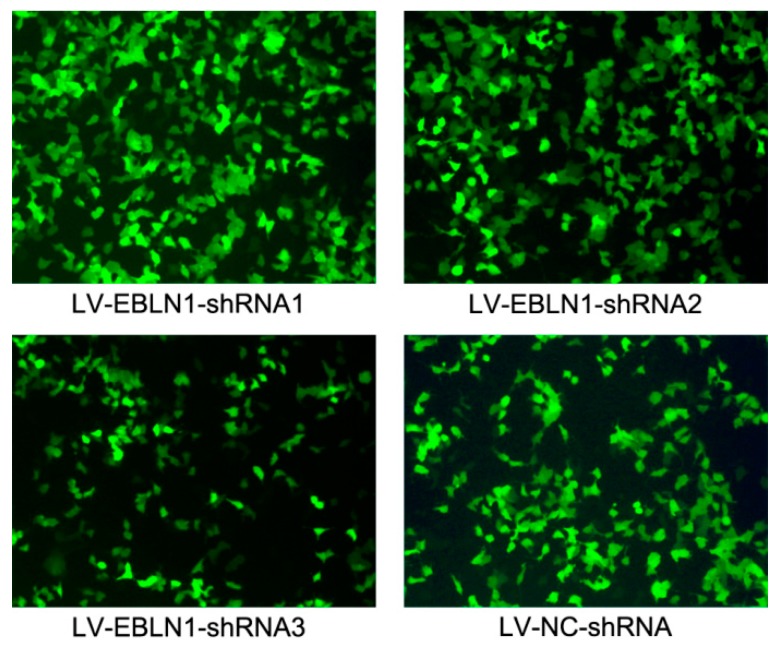
Examination of lentivirus infection efficiencies in oligodendroglia (OL) cells by fluorescence microscopy at 72 h post-infection (100×).

To explore the biological roles of *EBLN1* in human OL cells, three target-specific *EBLN1* shRNA expressing lentivirus and a negative-control shRNA expressing lentivirus were generated. After a 96-h lentivirus infection, EGFP (enhanced green fluorescent protein)-positive OL cells in each group were counted under a fluorescence microscope to determine the infection efficiencies. Those were 93.6%, 94.0%, 92.4%, and 95.0% in LV (lentivirus)-EBLN1-shRNA1, 2, 3, and LV-NC-shRNA group, respectively (Figure 1).

**Figure 2:**
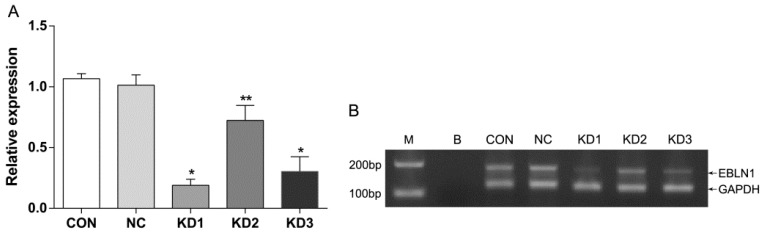
Determining the RNA interference efficiency of the LV (lentivirus)-EBLN1-shRNA vector in OL. (**A**) Relative expression of *EBLN1* detected by quantitative reverse transcription polymerase chain reaction (qRT-PCR); (**B**) the electrophoresis of qRT-PCR products. CON (control), uninfected group; NC (negtive control), LV-NC-shRNA group; KD (Knockdown) 1, LV-EBLN1-shRNA1 group; KD2, LV-EBLN1-shRNA2 group; KD3, LV-EBLN1-shRNA3 group. M, DL2000 Maker; B, blank control. * *p* < 0.001 *vs*. NC, ** *p* < 0.05 *vs*. NC.

For lacking of the evidence of EBLN1 protein expression, we only detected *EBLN1* mRNA expression in OL cells by RT-qPCR to determine the interference efficiency. Compared with the LV-NC-shRNA group, *EBLN1* mRNA expressions in three LV-EBLN1-shRNA groups were reduced by 81% (*p* < 0.001), 28% (*p* < 0.05), and 70% (*p* < 0.001), respectively. In addition, *EBLN1* mRNA expression was comparable between the LV-NC-shRNA group and the uninfected group (*p* > 0.05) (Figure 2A). The electrophoresis of quantitative reverse transcription polymerase chain reaction (qRT-PCR) products further confirmed that *EBLN1* mRNA was highly expressed in OL cells, which is comparable to GAPDH (glyceraldehyde-3-phosphate dehydrogenase), and LV-EBLN1-shRNA could markedly suppress *EBLN1* (Figure 2B). Thus, LV-EBLN1-shRNA1 was the most effective lentivirus for *EBLN1* silencing in OL cells, and the interference effects were specific to *EBLN1*. Therefore, LV-EBLN1-shRNA1 was used in the *EBLN1* knockdown group in the subsequent experiments.

### 2.2. EBLN1 Silencing Inhibits Oligodendroglia (OL) Cell Proliferation

**Figure 3:**
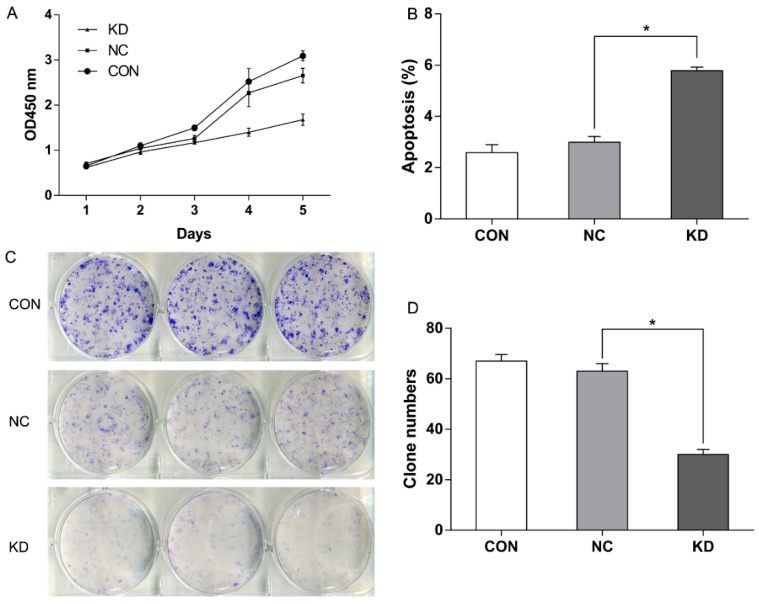
*EBLN1* silencing affects the proliferation, apoptosis, and colony formation of OL cells. (**A**) Growth curves of OL cells in 3 different groups, as measured by the CCK-8; (**B**) *EBLN1* gene silencing induced marked apoptosis in OL cells. (**C** + **D**) *EBLN1* gene silencing inhibited colony formation with OL cells. CON, uninfected group; NC, LV-NC-shRNA group; KD, LV-EBLN1-shRNA group. * *p* < 0.01.

To test the effects of *EBLN1* knock-down on proliferation, CCK-8 (Cell Counting Kit-8) assays were performed. The results showed that cell growth was significantly inhibited in the LV-EBLN1-shRNA group, compared with control and LV-NC-shRNA groups. A significant reduction of cell proliferation was observed in the LV-EBLN1-shRNA group at 72-h post-inoculation (about 26%). The inhibition efficiency became more evident (up to 84%) at 5 days post-inoculation (Figure 3A; *p* < 0.001). Meanwhile, the expression of *EBLN1* was reduced by 86% at 5 days post-inoculation.

### 2.3. EBLN1 Silencing Induces Apoptosis and Inhibits Colony Formation of OL Cells

To determine the effects of *EBLN1* gene silencing on apoptosis in OL cells, flow cytometry was performed with annexin V-APC (allophycocyanine) staining at 96-h post-inoculation. Our results showed that the percentage of apoptotic OL cells significantly increased in the LV-EBLN1-shRNA group (5.783 ± 0.138), compared with the LV-NC-shRNA (2.99 ± 0.232) and control groups (2.583 ± 0.313) (Figure 3B; *p* < 0.001). Meanwhile, the expression of *EBLN1* was reduced by 81% at 5 days post-inoculation.

Colony-formation assays were conducted to gain insight into the long-term effects of *EBLN1* silencing on cell proliferation. OL cells in each group were incubated for 14 days in 6-well plates and then the colony numbers were counted. The numbers of colony were 67 ± 2.65 in uninfected group, 63 ± 3.00 in LV-NC-shRNA group, and 30 ± 2.00 in LV-EBLN1-shRNA group. There were no significant differences between LV-NC-shRNA and control groups (*p* > 0.05). However, the colony-forming efficiency of the *EBLN1* knockdown OL cells was markedly less than that of OL cells in the LV-NC-shRNA and control groups (Figure 3C,D; *p* < 0.001).

### 2.4. EBLN1 Silencing Induces G2/M Phase Arrest in OL Cells

**Figure 4:**
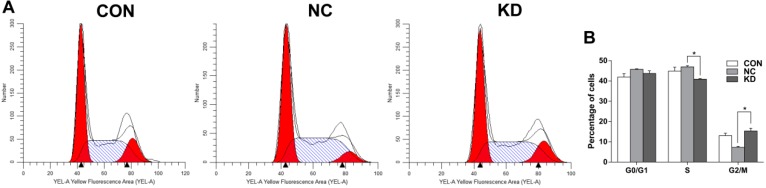
Flow cytometric detection of G2/M phase arrest induced in OL cells following *EBLN1* gene silencing. (**A**) Cell cycle distribution of OL cells were determined by using flow cytometry. The left red area is the G0/G1 phase, the right area is the G2/M phase, and the middle strip area is the S phase. (**B**) Knockdown of *EBLN1* induces G2/M phase arrest in OL cells. CON, uninfected group; NC, LV-NC-shRNA group; KD, LV-EBLN1-shRNA group. * *p* < 0.001.

To determine the effects of *EBLN1* silencing on cell-cycle control OL cells, a flow cytometry assay was performed when the expression of *EBLN1* was reduced by 81% at 96-h post-inoculation. Compared with the LV-NC-shRNA group, the proportion of cells in S phase significantly decreased in the LV-EBLN1-shRNA group (*p* < 0.01), but that in G2/M phase significantly increased (*p* < 0.01) (Figure 4).

### 2.5. EBLN1 Silencing Has No Effect on the Migration of OL Cells

**Figure 5:**
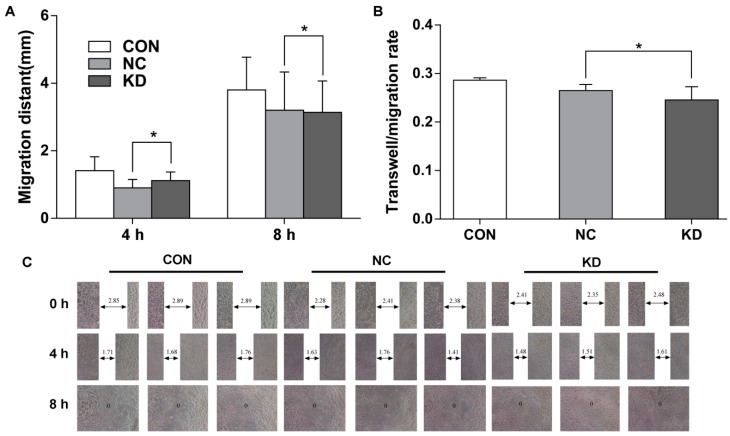
Suppression of *EBLN1* expression does not affect the migration of OL cells. (**A**) Statistical analysis of the results of wound-scratch assays of OL cells in the 3 indicated groups; (**B**,**C**) effects of *EBLN1* silencing on the transwell migration ability of OL cells, as determined in wound-healing assay. CON, uninfected group; NC, LV-NC-shRNA group; KD, LV-EBLN1-shRNA group. * *p* > 0.05.

At 4 days after lentivirus infection, when the expression of *EBLN1* was reduced by 81%, wound-scratch assays were performed to test cell migration capability. Our results demonstrated the wound-closure rates did not differ significantly between the LV-EBLN-shRNA and LV-NC-shRNA groups at 4 and 8 h after scratching (*p* > 0.05; Figure 5A,C).

Transwell migration assay results showed that the migration rates in the LV-EBLN1-shRNA, LV-NC-shRNA, and uninfected groups were 0.246 ± 0.028, 0.265 ± 0.013, and 0.286 ± 0.005, respectively. No significant differences were observed between these groups (*p* > 0.05; Figure 5B).

### 2.6. EBLN1 Silencing Changes Gene Expression Profiles in OL Cells

To determine the effects of *EBLN1* silencing on gene expression in OL cells, cDNA microarray analysis was performed at 4 days after lentivirus infection, when the expression of *EBLN1* was reduced by 82%. We observed 1067 upregulated and 2004 downregulated genes in the LV-EBLN1-shRNA group, compared with LV-NC-shRNA group. The top 100 most-changed genes were listed in Supplementary Materials Table S1. Cluster analysis revealed that the top 10 most upregulated genes were *PI3*, *RND3*, *BLZF1*, *SOD2*, *EPGN*, *SBSN*, *INSIG1*, *OSMR*, *CREB3L2*, and *MSMO1*, while the top 10 most downregulated genes were *KRTAP2-4*, *FLRT2*, *DIDO1*, *FAT4*, *ESCO2*, *ZNF804A*, *SUV420H1*, *ZC3H4*, *YAE1D1*, and *NCOA5*.

**Figure 6:**
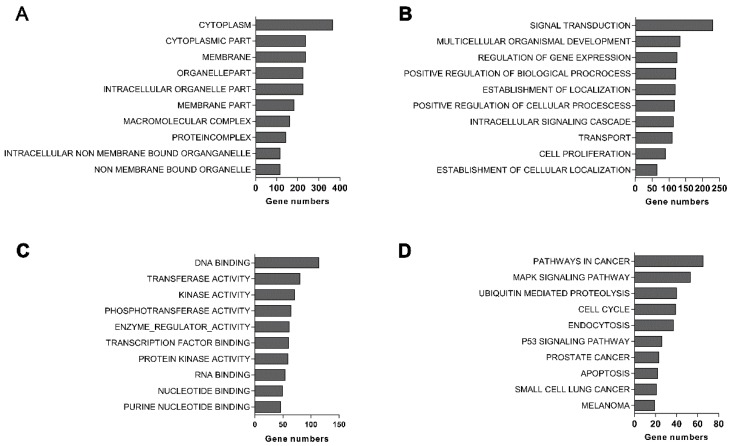
GO and pathway analysis of differentially expressed genes. (**A**) The main cellular component of differentially expressed genes by GO analysis; (**B**) the main biological processes of differentially expressed genes by GO analysis; (**C**) the main molecular functions of differentially expressed genes identified by GO analysis; and (**D**) pathway analysis of differentially expressed genes.

Gene ontology analysis showed that most of differential genes were composed of cytoplasm, memerane, intracellular organelle (Figure 6A). The biological processes regulated by these differential genes were mainly associated with multicellular organismal development, signal transduction, intracellular signaling cascades, and cell proliferation (Figure 6B). Their molecular functions were mainly involved in transferase activity, enzyme regulator activity, DNA binding, phosphotransferase activity, kinase activity, and transcription factor binding (Figure 6C). Pathway analysis demonstrated that they were mainly related to cell cycle, ubiquitin hydrolysis, mitogen-activated protein kinase (MAPK), p53, and apoptosis pathways (Figure 6D).

### 2.7. Verification of Differential Genes by Quantitative Reverse Transcription Polymerase Chain Reaction (qRT-PCR)

**Figure 7:**
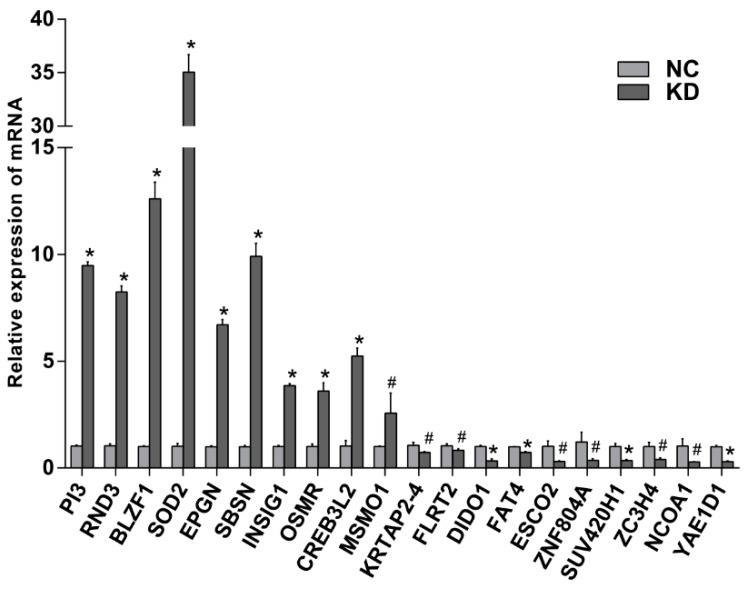
The mRNA expression levels of the top 20 most changed genes by qRT-PCR. NC, LV-NC-shRNA group; KD, LV-EBLN1-shRNA group. * *p* < 0.001, ^#^ *p* < 0.05.

To validate the results of cDNA array, qRT-PCR was performed to detect the expressions of the top 20 most changed genes. GDPAH served as an endogenous control. As shown in Figure 7, the results of qRT-PCR were consistent with the gene expression profiles and confirmed that the microarray data were reliable.

Three genes *RND3*, *OSRM*, and *CREB3L2* were focused, for their closely relation to glioma. Compared with LV-NC-shRNA group, their relative expressions in *EBLN1* knockdown OL cells were 8.24 ± 0.29, 3.60 ± 0.39, 5.25 ± 0.37, respectively (Figure 7).

## 3. Discussion

BDV is an ancient neurotropic virus and the etiological agent of fatal encephalitis in horses and sheep. BDV is characterized by persistent infection in the nervous system of many animals. In experimental animals, such as rats and mice, BDV can induce cognitive deficiencies and behavioral alterations [26,27,28]. Epidemiologic studies have demonstrated that BDV can infect healthy humans and is possibly associated with some neuropsychiatric disorders including bipolar depression and schizophrenia [13,15,29,30,31]. The discovery of *EBLNs* in the human genome further confirmed the closely relationship between BDV and humans. Thus far, the expression at transcriptional level of all seven human *EBLNs* has been confirmed. However, the functions of human *EBLNs* are yet not clear. Seeing that HERVs are involved in some human diseases, including cancers, such as endogenous retroviral LTR, K, and Fc-1 [6,32,33], here we investigated the biological functions of *EBLN1* in human OL cells. The OL cells used in this study were permanent cell line from human fetal brain (details in Section 4.1).

The expression level of *EBLN1* in OL cells was firstly confirmed by qRT-PCR, although previous reports demonstrated that *EBLN1* was detected in OL cells [8,25]. Our results showed that *EBLN1* was highly expressed in OL cells, almost at the same level as GAPDH. Then, we constructed three lentiviral vectors expressing *EBLN1* shRNAs and tested their inhibition efficiencies. Due to lack of evidence that the EBLN1 protein is expressed, we only measured the inhibition efficiencies at *EBLN1* mRNA levels by qRT-PCR. Compared to the negative control, LV-EBLN1-shRNA1 lentiviral vectors could most efficiently reduce the expression of *EBLN1* mRNA in OL cells by 81% after a 96-h infection. Therefore, LV-EBLN1-shRNA1 lentiviral vectors were used in subsequent experiments.

Previous studies demonstrated BDV had diverse effects on cellular proliferation and apoptosis, depending on the virus isolate used and the host cell lines infected. BDV strain He/80 infection decelerated the proliferation of primary fibroblast cells from Lewis rats [20]. Moreover, BDV He/80 infection increased apoptosis of granule cell neurons in neonatal Lewis rats, but inhibited apoptosis of C6 rat astroglioma cells [34,35]. In addition, BDV strain Hu-H1, isolated from a bipolar patient by Bode in 1996 [11], inhibited cellular proliferation and promoted apoptosis in OL cells via Bax upregulation and Bcl-2 downregulation, contrary to laboratory BDV strain V [36].

Considering that *EBLN1* shares high identity with BDV *N* gene and N protein is an important BDV antigen, we focused on the effect of *EBLN1* on cellular proliferation and apoptosis in OL cells. Our results showed that the cellular proliferation significantly decreased from 72 h to 5 days after lentivirus infection, which was consistent with the results that the inhibit efficiency of LV-EBLN1-shRNA reached above 80% after a 96-h lentivirus infection. Moreover, the colony formation of *EBLN1* silencing OL cells was noticeably decreased. Cell cycle was measured at 96 h after lentivirus infection by flow cytometer. Compared to the LV-NC-shRNA group, the OL cells in S-phase significantly decreased, but the cells in G2/M phase significantly increased in the LV-EBLN1-shRNA group. Taken together the results of cell proliferation, our results showed that *EBLN1* silencing could induce G2/M phase arrest in OL cells. To confirm this result, cell cycle assay in our next works should be taken after synchronization by inducing G2/M or S arrest. Moreover, the apoptosis of OL cell was tested after a 96-h lentivirus infection. The results displayed that *EBLN1* silencing increased apoptosis, which was consistent with our results that the inhibit efficiency of LV-EBLN1-shRNA reached over 80% after a 96-h lentivirus infection, and the cell growth of *EBLN1* silencing OL cells was decreased at 4 days post-infection. However, the migration abilities of OL cells in both wound-scratch and transwell migration assays were not significantly changed by *EBLN1* silencing. Sine these migration assays might be affected by the cell death, these results of migration assays should be further evaluated in our future works.

To explore the mechanism underlying these findings, we further investigated differentially expressed genes in *EBLN1* knockdown OL cells by microarray analysis. Our results revealed that 3071 genes were dysregulated in OL cells after *EBLN1* silencing, which suggested *EBLN*1 was a key gene involved in multi-functions in OL cells. Pathway analysis demonstrated that these differentially expressed genes mainly affect cell-cycle progression, apoptosis, MAPK, and p53 signaling pathways, which was supported by the alterations in proliferation and apoptosis of *EBLN1* knockdown OL cells. qRT-PCR was performed to validate the results of a cDNA array. The mRNA expressions of the top 20 most-changed genes were consistent with the gene expression profiles, which confirmed that the microarray data were reliable.

Some of these most-changed genes were closely related to glioma, such as *RND3*, *OSMR*, and *CREB3L2*. These genes were frequently dysregulated in glioma cells and played important roles in regulating cell growth and increased apoptosis. We presumed that these three genes might be the key factors in *EBLN1* regulating the proliferation and apoptosis of OL cells.

RND3 (also named RhoE) is an atypical member of the Rho GTPase family. RND3 inhibits Rho kinase-mediated biological functions including actin cytoskeleton formation, cell transformation, proliferation, and apoptosis. The role of RhoE in cancer is currently controversial [37,38,39]. In human glioblastoma, RND3 expression was found to be significantly decreased, which caused increased Notch-pathway activity and enhanced glioma cell proliferation [40]. RND3 functioned as a negative regulator of the Notch pathway by promoting the ubiquitination and degradation of Notch transcriptional complex [40]. In this study, *RND3* was upregulated after *EBLN1* silencing in OL cells, and *MAML2*, coded a coactivator for Notch protein, was downregulated. This suggested that one of the mechanisms of *EBLN1* regulating the proliferation and apoptosis of OL cells might be RND3 induced repression of Notch-pathway.

The oncostatin M receptor (OSMR) gene encodes a subunit of the type-II receptor for oncostatin M (OSM). OSM is an IL-6 family cytokine that is associated with multiple biological processes and cellular responses including growth, differentiation, and inflammation [41]. High expression of OSMR was observed in glioblastoma, especially in the mesenchymal subtype, and was regarded as a prognostic risk factor. OSM-OSMR signaling regulated the pathological progression of glioma via STAT3, a key transcription factor involved in the Janus kinase-signal transducer and activator of transcription (JAK/STAT) signaling pathway, which regulates the expression of genes involved in diverse functions such apoptosis, proliferation, and differentiation [42]. Unlike with glioblastoma, *OSRM* was upregulated after *EBLN1* silencing in OL cells. Meanwhile, we found some genes in the JAK/STAT signaling pathway were downregulated, such as *STAT6* and its reactor *NCOA1*, and anti-apoptotic proteins (Bcl-2 and MCL1). Therefore, the effects of *EBLN1* on cell proliferation and apoptosis might be related with the downregulation of anti-apoptotic proteins through JAK/STAT pathway, special STAT6, regulated by OSM-OSMR signaling.

CREB3L2 (also known as BBF2H7), an endoplasmic reticulum stress transducer, belongs to the cAMP responsive element-binding (CREB)/activating transcription factor (ATF) family. ATF5, another member of the CREB/ATF family, is a target gene of CREB3L2. Previous findings demonstrated that CREB3L2 could suppress apoptosis via the ATF5-MCL1 pathway in growth plate cartilage [43]. ATF5 is highly expressed in primary brain tumors, especially in glioblastoma, and plays a key role in promoting cancer cell survival through the CREB3L2/ATF5/MCL1 pathway. In this pathway, induction of CREB3L2 by a RAS (renin–angiotensin system)/MAPK or phosphatidylinositol 3-kinase (PI3K) signaling pathway can activate ATF5, which promotes survival by stimulating transcription of the anti-apoptotic MCL1 (myeloid cell leukemia 1 protein) protein [44]. In our work, the expression of *ATF5* had no statistical differences between the *EBLN1* silencing group and negative control group. However, other members of the CREB/ATF family including *ATF2* and *ATF3* were downregulated. Though the relation of upregulated *CREB3L2* and downregulated *ATF2* and *ATF3* was unknown, the effects of *EBLN1* silencing on cell proliferation and apoptosis might be explained by the low expressions of *ATF2*, *ATF3* and *MCL1*.

## 4. Experimental Section

### 4.1. Cells and Culture

The human OL cell line [35] was kindly provided by Hanns of the Berlin Free University, Berlin, Germany and Liv Bode, Robert Koch Institute, Berlin, Germany. OL cells were permanent human oligodendroglia cell line, established by Y. Iwasaki from human fetal brain at the Wistar Institute, PA, USA, and then allocated to Hanns Ludwig in the early 1980s. OL cells were cultured in Dulbecco’s Modified Eagle’s Medium (DMEM, Invitrogen, Grand Island, NY, USA) with high glucose (4.5 g/L, Gibco, Grand Island, NY, USA), supplemented with 1% penicillin, 1% streptomycin (Sigma, Shanghai, China), and 5% heat inactivated fetal bovine serum (FBS, Gibco, Grand Island, NY, USA). Cells were incubated at 37 °C in a 5% CO_2_ incubator.

### 4.2. Construction of an EBLN1 shRNA-Expressing Lentiviral Vector and Infection

We designed *EBLN1* shRNA and negative control shRNAs based on the mRNA sequence of human *EBLN1* (NM_001199938). The three target-specific sequence of *EBLN1* shRNA was: 5′-GCCTCCAAGTCAGAGACA-3′ (shRNA1); 5′-GCTATAGATTGGATCAACT-3′ (shRNA2); 5′-GCAACTAAGTCTATGCTAG-3′ (shRNA3). The sequence of the negative-control shRNA was 5′-TTCTCCGAACGTGTCACGT-3′. Lentivirus was packaged in 293T cells by co-infection with the modified pGV277-EGFP viral vector and the pHelper 1.0 and pHelper 2.0 helper plasmids (GeneChem Technology, Shanghai, China). The *EBLN1* shRNA-expressing lentiviral vectors were named LV-EBLN1-shRNA1, LV-EBLN1-shRNA2, and LV-EBLN1-shRNA3, respectively, and the negative control lentiviral vector was named LV-NC-shRNA.

OL cells were divided into 3 groups: uninfected group (CON), LV-NC-shRNA group (NC), and LV-EBLN1-shRNA group (KD). OL cells infected for 16 h with LV-EBLN1-shRNA or LV-NC-shRNA in enhanced infection solution at a multiplicity of infection of 2 and subsequently placed in fresh medium. After 72 h, the fluorescence of OL cells was measured and the infection efficiency was calculated as the ratio of fluorescent OL cells to all OL cells.

### 4.3. Detection of EBLN1 mRNA Expression

Quantitative reverse transcription polymerase chain reaction (qRT-PCR) experiments were performed to detect *EBLN1* mRNA expression. Total RNA was extracted from OL cells in each group with the Trizol reagent (Invitrogen) and quantified with a NanoDrop spectrophotometer (NanoDrop Technologies, Wilmington, DE, USA). To avoid the presence of contaminating DNA, total RNA was treated with DNase (Invitrogen) according to manufacturer’s instructions, followed by reverse transcription into cDNA (Takara, Toyoto, Japan). The sequences of primers used to amplify *EBLN1* cDNA were as follows: 5′-ACCTAGCAACAGCAGCAAACTA-3′ (forward) and 5′-CAAATCCCGAAATCCCATAAC-3′ (reverse). The sequences of primers used to amplify the reference gene GAPDH were as follows: 5′-GGTCTCCTCTGACTTCAACA-3′ (forward) and 5′-AGCCAAATTCGTTGTCATAC-3′ (reverse). PCR were performed using 2 µL of cDNA in a Corbett Research Rotor-Gene 6000 Thermocycler (Corbett Research, Mortlake, Australia) in 25 µL reaction mixtures. Thermocycling conditions consisted of an initial denaturation step for 10 min at 94 °C, followed by 40 cycles of 94 °C for 30 s and 56 °C for 45 s. PCR was repeated in 3 independent experiments. Relative expression levels of *EBLN1* mRNA were normalized against that of GAPDH, using the 2^−ΔΔ*C*t^ method. Meanwhile, 5 µL PCR products of were electrophoresed on 2% agarose gels.

### 4.4. Cell Proliferation Analysis

Cell proliferation was analyzed using the Cell Counting Kit-8 (CCK8; Beyotime, Shanghai, China). OL cells were plated in each well of 96-well plates at a density of 2000 cells in 100 µL culture medium. At the same time, OL cells in LV-EBLN1-shRNA group were infected with lentivirus. After various incubation periods ranging from 1 to 5 days, 10 µL of CCK-8 solution was added the cells and incubated for 2 h at 37 °C. The absorbance was measured at 450 nm with an ultraviolet spectrometer (Bio-Rad, Shanghai, China). The experiments were performed in quadruplicate and repeated in triplicate.

### 4.5. Analysis of Apoptosis

Cell apoptosis was measured by annexin V-APC staining. After 96-h incubation, infected cells were washed twice with PBS and resuspended in binding buffer at a density of 5 × 10^5^ cells/mL, followed by the addition of 10 µL Annexin V-APC. After gentle mixing, the cells were incubated for 15 min at room temperature in the dark. The cells were analyzed by flow cytometry within 1 h.

### 4.6. Colony-Formation Assay

After 96-h of lentivirus infection, 800 OL cells in each group were plated into each well of 6-well plates and cultured for 14 days at 37 °C in a 5% CO_2_ incubator. Then, cells were rinsed twice with PBS and fixed in l mL of paraformaldehyde for 30 min. The cells were stained with Giemsa stain for 20 min and then washed with ddH_2_O. The plates were dried at room temperature and colonies containing more than 50 cells were counted under a light microscope.

### 4.7. Cell Cycle Analysis

After 96-h of lentivirus infection, OL cells (1 × 10^6^) in each group were washed with cold phosphate-buffered saline (PBS), fixed in 70% ethanol at 4 °C for at least 2 h, and stained with 0.5 mL propidium iodide solution for 30 min in the dark. Measurements of DNA contents and cell-cycle analysis were performed by flow cytometry (BD Biosciences, San Jose, CA, USA). All experiments were repeated in 3 independent experiments.

### 4.8. In Vitro Wound-Healing Assay

After a 96-h lentivirus infection, 3 × 10^4^ OL cells were seeded into 96-well plates and grown at 37 °C in a 5% CO_2_ incubator. When the confluence reached 90%, the medium was removed, and a wound in the monolayer was made using a pipette tip, followed by washing with PBS thrice to remove the non-adherent cells. The wound area was photographed immediately after wounding and at 0, 4, and 8 h post-wounding. The width of the wound was measured, and the migration rates were calculated.

### 4.9. Transwell-Migration Assay

After a 96-h lentivirus infection, transwell-migration assay was performed. Transwell chambers were placed in plates, and 100 µL of serum-free medium was added into the upper chambers. After incubation at 37 °C for 2 h, the medium was removed. Subsequently, 1 × 10^5^ infected cells were re-suspended in 100 µL serum-free medium and added to the upper chambers, and medium containing 30% FBS was added to the lower chambers, followed by incubation at 37 °C in a CO_2_ incubator for 24 h. After removing the non-migrated cells, the upper chambers were stained with Giemsa for 20 min, after which the migrated cells were observed under a microscope and resolved in 10% acetic acid. Then, the absorbance (optical density) at 570 nm (OD_570_ nm) was measured. Meanwhile, 5000 infected OL cells were incubated in 96-well plates at 37 °C for 4 h. After 3-(4,5-dimethylthiazol-2-yl)-2,5-diphenyltetrazolium bromide (MTT) stain, the absorbance at 490 nm (OD_490_ nm) was measured with a spectrophotometer. The migration rate was calculated by using the following equation: migrating rate (%) = OD_570 nm_/OD_490 nm_.

### 4.10. Microarray Analysis

The cDNA microarray was analyzed using GeneChem Technology (Shanghai, China). Briefly, total RNA was extracted from OL cells after a 96-h lentivirus infection, then cDNA was synthesized and transcribed into biotin-labeled sRNA using the GeneChip 3′ IVT Express Kit. Subsequently, the sRNA was hybridized to a PrimeView™ Human Gene Expression Array Plate (Affymetrix, Shanghai, China), which was scanned using a GeneChip Scanner 3000. The differently regulated genes with absolute fold-change values ≥2 and *p-*value ≤0.05 were used for subsequent gene ontology and pathway analysis.

### 4.11. Verification of Differential Genes by Quantitative Reverse Transcription Polymerase Chain Reaction (qRT-PCR)

The top 20-most changed genes were elected and verified by qRT-PCR using the primers listed in Supplementary Materials Table S2. The primers for GAPDH (Glyceraldehyde-3-phosphate dehydrogenase) and the reaction conditions of qRT-PCR were described as in Section 4.3, except the annealing temperature is 58 °C.

### 4.12. Statistical Analysis

Statistical analysis was performed with SPSS software, version 19.0 (IBM Corporation, New York city, NY, USA). Qualitative data were expressed as the mean ± SD. Differences between groups were analyzed with Student’s *t* test. A value of *p* < 0.05 was considered statistically significant.

## 5. Conclusions

In summary, we focused on the cellular biological functions of *EBLN1* in human OL cells. Knock-down of *EBLN1* by lentivirus-mediated shRNA suppressed proliferation and induced apoptosis. Numerous genes were dysregulated by *EBLN1* silencing, some of which may be key target genes of *EBLN1*, such as RND3, OSMR, and CREB3L2. Our work provides meaningful data and offers a new direction for further studies on *EBLN1*. Though we proposed the possible mechanisms involved in the function of *EBLN1*, more work will be done to confirm them or discover other underlying molecular mechanisms in future studies.

## Supplementary Materials

Supplementary materials can be found at http://www.mdpi.com/1422-0067/17/4/435/s1.
