# Supplementary material for: Knock-Down of Endogenous Bornavirus-Like Nucleoprotein 1 Inhibits Cell Growth and Induces Apoptosis in Human Oligodendroglia Cells
Source: Int J Mol Sci. 2016 Mar 24;17(4):435. doi: 10.3390/ijms17040435 (PMC4848891; doi:10.3390/ijms17040435)
Supplement: Supplementary file 1 [file ijms-17-00435-s001.pdf]

Supplementary Materials: Knock-Down of Endogenous Bornavirus-Like Nucleoprotein 1 Inhibits Cell Growth and Induces Apoptosis in Human Oligodendroglia Cells

Peng He, Lin Sun, Dan Zhu, Hong Zhang, Liang Zhang, Yujie Guo, Siwen Liu, Jingjing Zhou, Xiaoyan Xu and Peng Xie

Table S1. Top 100 most-changed genes between LV-EBLN1-shRNA and LV-NC-shRNA groups.

Regulation	Gene Symbol
Down	*KRTAP2-4, FLRT2, DIDO1, FAT4, ESCO2, ZNF804A, SUV420H1, ZC3H4, YAE1D1, NCOA5, ESM1, INTS7, RIMS2, TRAK2, STXBP5, BTBD7, RIN2, PRMT6, DNAJB6, CEP19, BRI3BP, HAUS6, CSTF3, ZNF480, GHR, THEM4, FAM111B, ZNF75A, CHAC2, TRIM2, NRP1, C3orf23, C7orf49, ZNF789, MOCS3, MBLAC2, ZNF189, ADAMTS5, ZNF252P, LARP4, HIST1H2BD, SFPQ, DNAJC11, IFNAR1, PIGM, CSRP2BP, KCNMA1, ARL14, VANGL1, PCLO*
Up	*PI3, RND3, BLZF1, LOC100129518, EPGN, SBSN, INSIG1, OSMR, CREB3L2, MSMO1, FBN2, SCD, C15orf48, ATF7IP, ARG2, SFT2D3, SAT1, ATF3, LOC100287223, CFB, GATSL3, THBD, CCL20, DHDH, HBP1, CLK1, RPL28, DUSP16, C3, KLHL24, DDIT3, GBP2, KLF3, C1R, PER1, GEM, LDLR, KLF9, ANKHD1, CDC16, IGF2, CDKN1C, HMGCS1, ZFP36, BBC3, PTGES, TMEM41B, RAB18, LOC100506403, SIK1*

Table S2. Primers of qRT-PCR for Top 20 most-changed genes.

Gene Name	Forward Primer (5′–3′)	Reverse Primer (5′–3′)
BLZF1	ACAGACAGAGGTAAATCGTGAGT	GGGCTAGACGTTCAAAGTGATAC
CREB3L2	GAGCCAGTTACAGACGAACCAC	TCTGGCACGAGGAATCAACC
DIDO1	CCAGAGTCAAAAGGCGTCGG	CGTGGAGTAAGCTCCTTCGC
EPGN	GGACAGTTAACAAAACAGAAGCTGA	AGCTCATGGTGGAATGCACA
ESCO2	GCTGGCTAGGCTGAGGAGAG	TCAGTGAAGTGTAAAAGGCTGTC
FAT4	CACCCTACTCAGGACTTCGG	CCATCAAAACCTGTTTGGGC
FLRT2	GCGGCGACCCCTAAAACAAT	CAATCTGCAATCTGACGGCTG
INSIG1	ATCCAGAGGAATGTCACTCTCTT	AGGGGTACAGTAGGCCAACAA
KRTAP2-4	ACAGAGCAATACACTGAAGCCT	GTGGGTGAGGGTGGTAATGG
MSMO1	GTTGGAACACCTGGCGAGT	CACAGCCAAGGATGCTGAACT
NCOA5	ATACGGCTCCATCAAGACCC	TGGGCTCTCTCCTTGGACTT
OSMR	CTATCTGAAATCCAAGGCGAGG	ATTCTGGCTTTAGGTTGTCCAC
PI3	CCGCTGCTTGAAAGATACTGACTGC	CCTGAATGGGAGGAAGAATGGAC
RND3	CTCTTACCCTGATTCGGATGCT	GATCAGACTTGCAGCCGACCA
SBSN	GCTGCTGAATGGCAACCATC	GATGAAAGGCGTGTTGACCG
SOD2	TTTCAATAAGGAACGGGGACAC	GTGCTCCCACACATCAATCC
SUV420H1	GGTGGTGAATGGCAGGAGAA	ACCATTACATCTGTTCGACCTCC
YAE1D1	ACTATGGACGACTCCGAGGAA	TCACACTGGCCAACTGCATC
ZC3H4	GAATGATGCCCCCTATCCCG	CTTCGTAGTGCCCGTAGTCC
ZNF804A	TTGTCATCAGCTCCACGCAT	CCTTCTCAGCATAGTCCAGAGT
